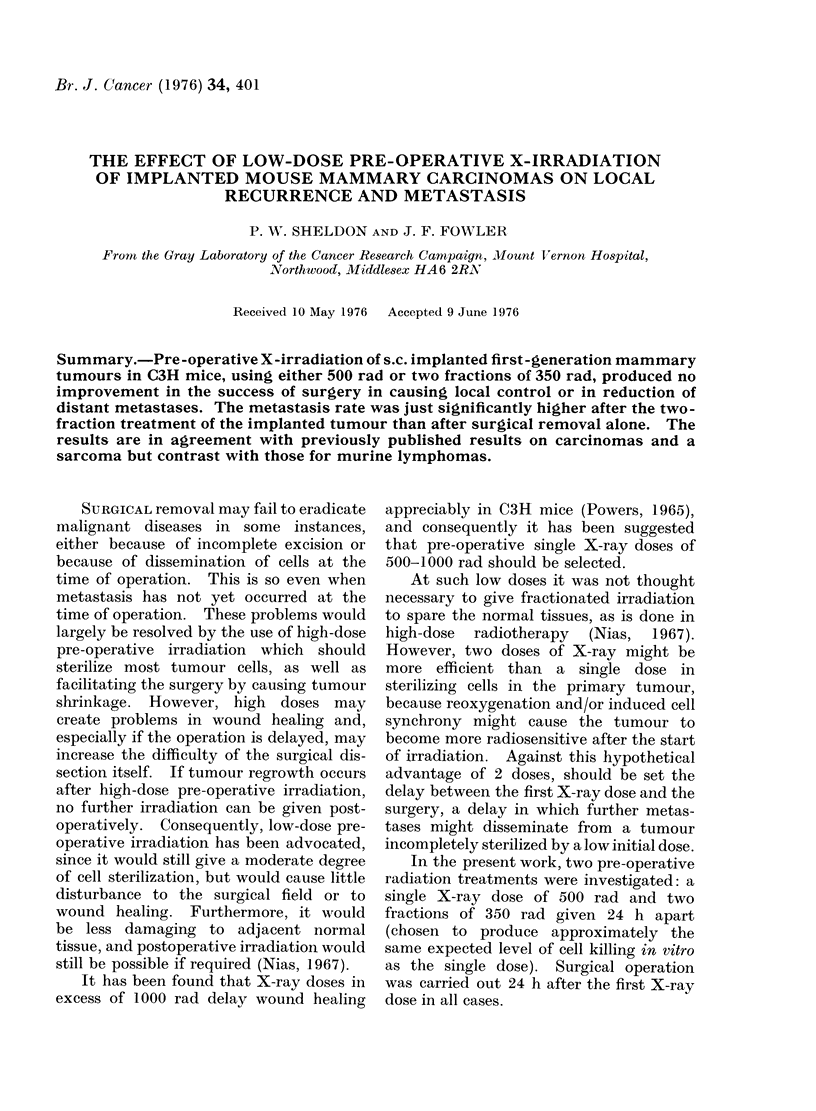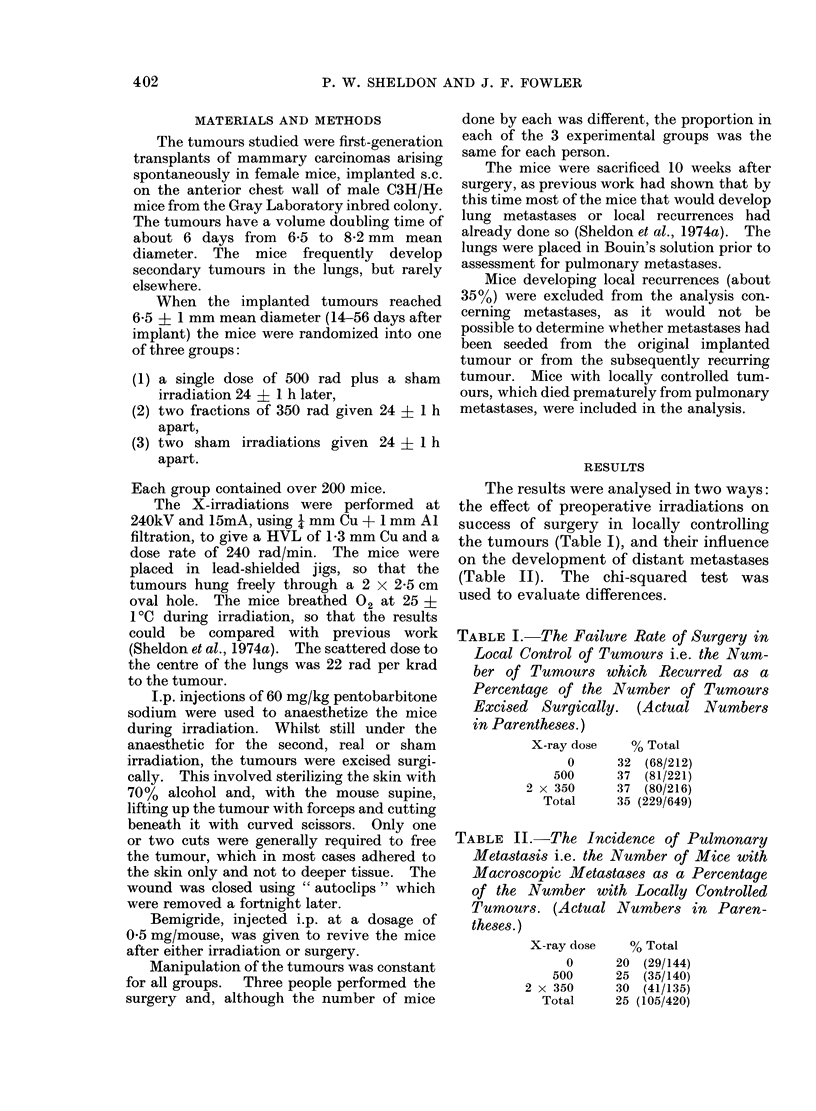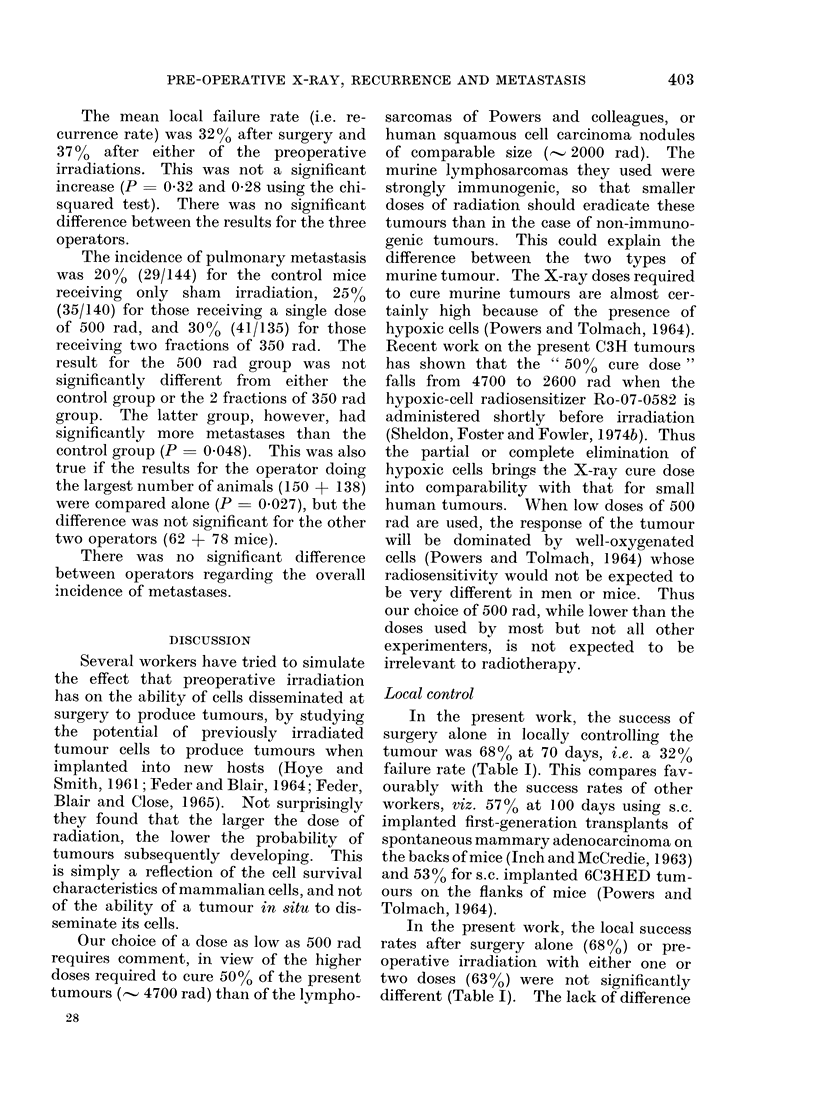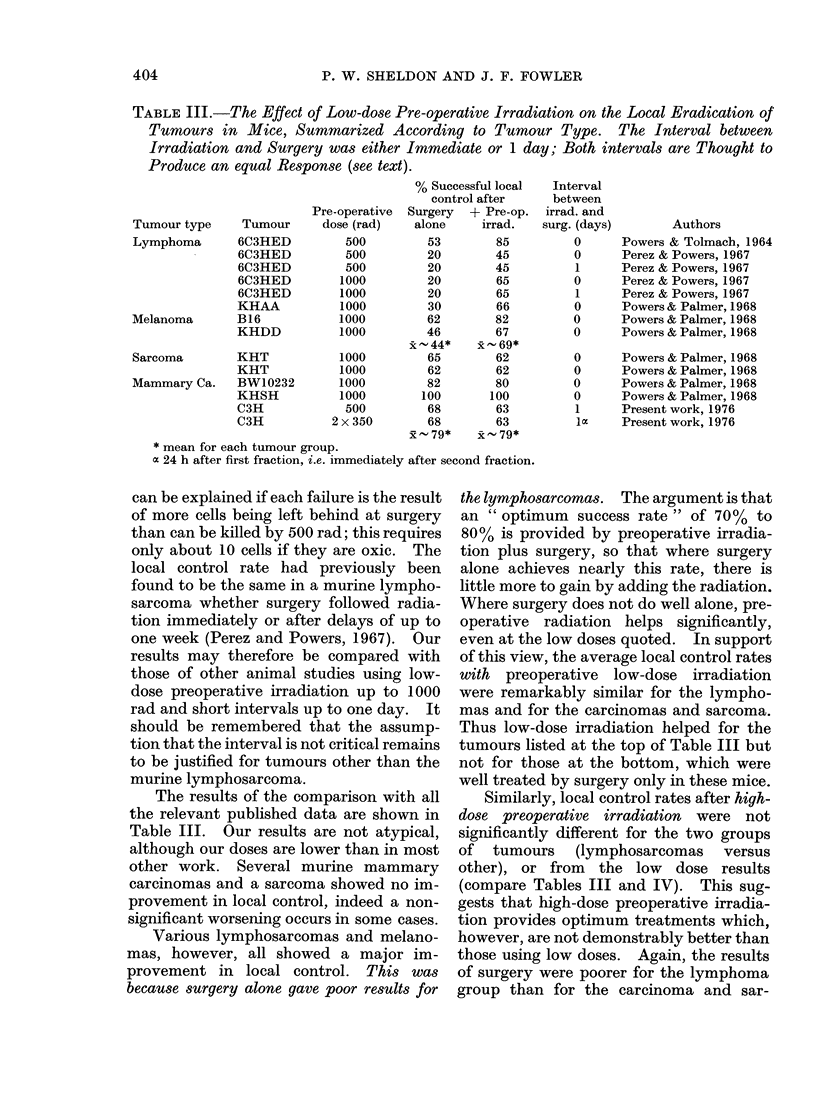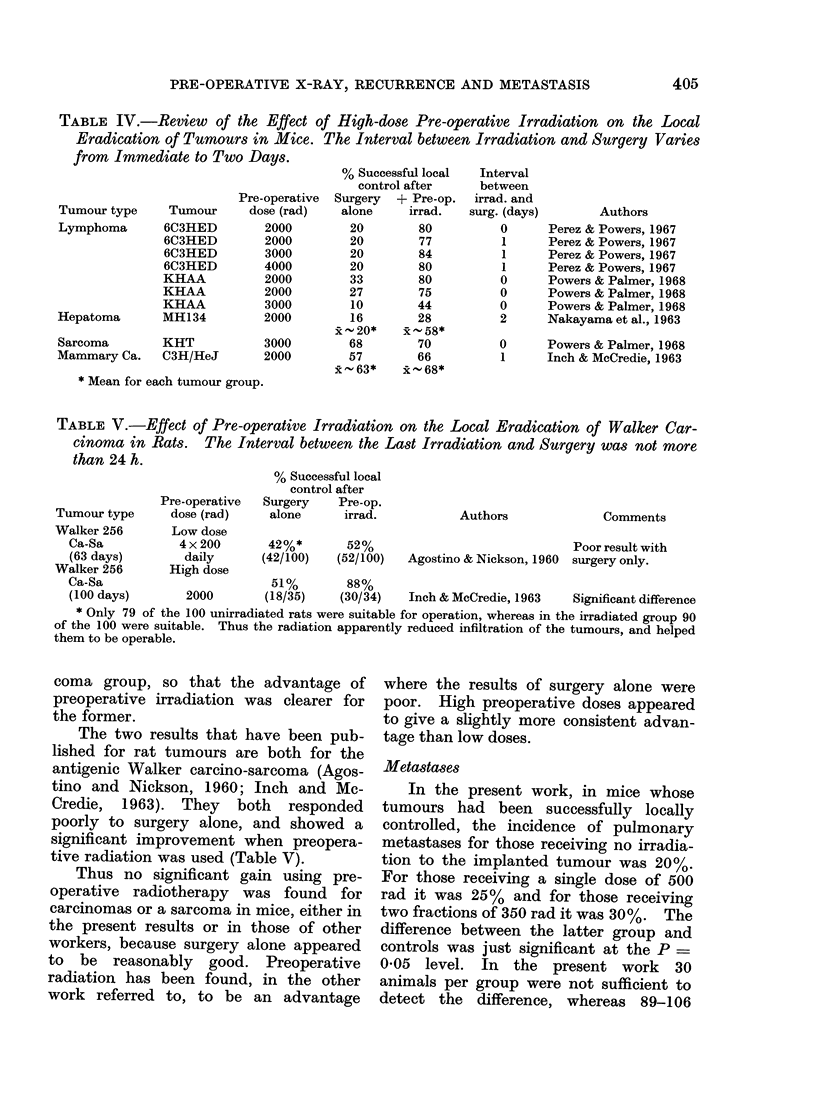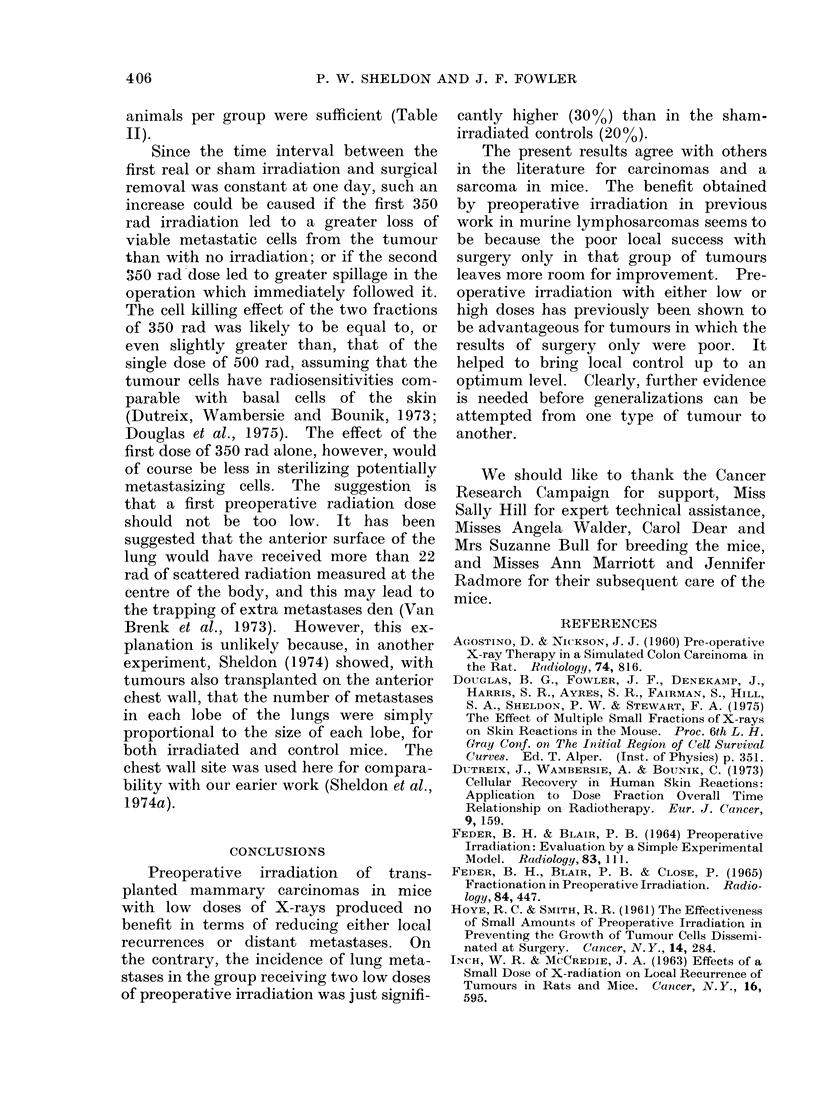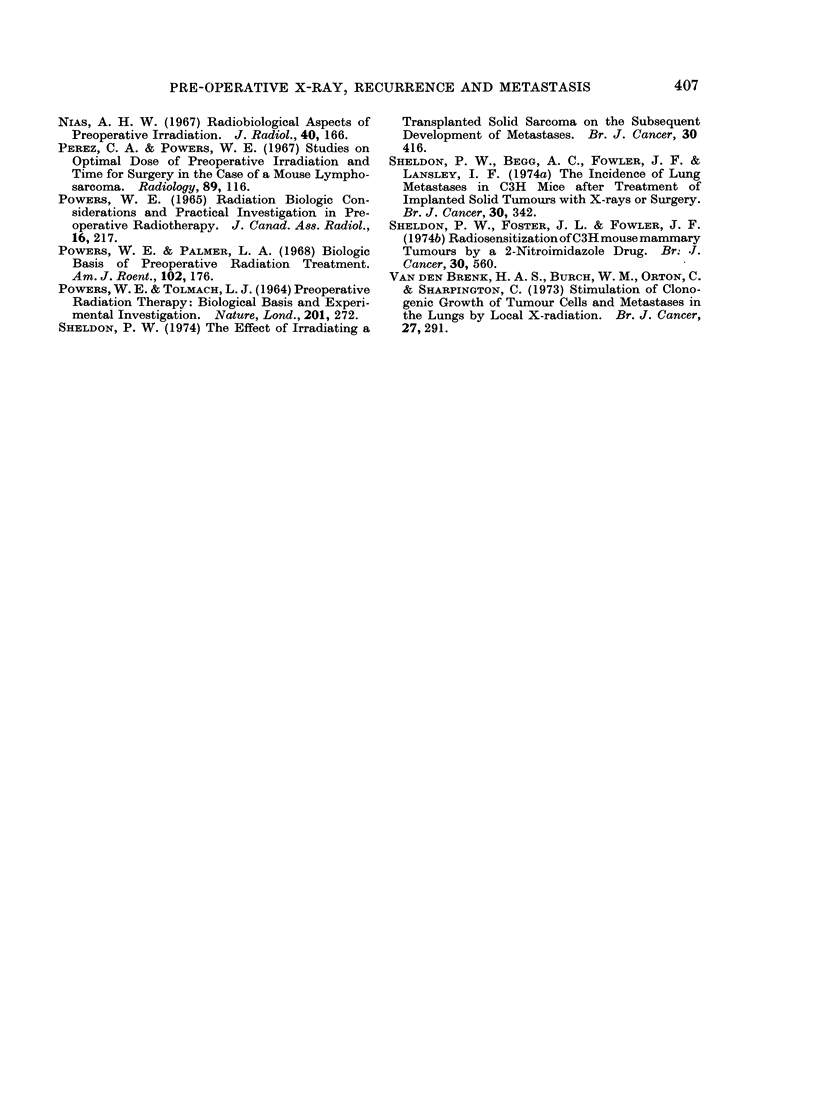# The effect of low-dose pre-operative X-irradiation of implanted mouse mammary carcinomas on local recurrence and metastasis.

**DOI:** 10.1038/bjc.1976.183

**Published:** 1976-10

**Authors:** P. W. Sheldon, J. F. Fowler

## Abstract

Pre-operative X-irradiation of s.c. implanted first-generation mammary tumours in C3H mice, using either 500 rad or two fractions of 350 rad, produced no improvement in the success of surgery in causing local control or in reduction of distant metastases. The metastasis rate was just significantly higher after the two-fraction treatment of the implanted tumour than after surgical removal alone. The results are in agreement with previously published results on carcinomas and a sarcoma but contrast with those for murine lymphomas.


*Br. J. Cancer* (1976) **34**, 401

### THE EFFECT OF LOW-DOSE PRE-OPERATIVE X-IRRADIATION OF IMPLANTED MOUSE MAMMARY CARCINOMAS ON LOCAL RECURRENCE AND METASTASIS

P. W. SHELDON AND J. F. FOWLER

*From the Gray Laboratory of the Cancer Research Campaign, Mount Vernon Hospital, Northwood, Middlesex HA6 2RN*

Received 10 May 1976 Accepted 9 June 1976

**Summary.—Pre-operative X-irradiation of s.c. implanted first-generation mammary tumours in C3H mice, using either 500 rad or two fractions of 350 rad, produced no improvement in the success of surgery in causing local control or in reduction of distant metastases. The metastasis rate was just significantly higher after the two-fraction treatment of the implanted tumour than after surgical removal alone. The results are in agreement with previously published results on carcinomas and a sarcoma but contrast with those for murine lymphomas.**

SURGICAL removal may fail to eradicate malignant diseases in some instances, either because of incomplete excision or because of dissemination of cells at the time of operation. This is so even when metastasis has not yet occurred at the time of operation. These problems would largely be resolved by the use of high-dose pre-operative irradiation which should sterilize most tumour cells, as well as facilitating the surgery by causing tumour shrinkage. However, high doses may create problems in wound healing and, especially if the operation is delayed, may increase the difficulty of the surgical dissection itself. If tumour regrowth occurs after high-dose pre-operative irradiation, no further irradiation can be given post-operatively. Consequently, low-dose pre-operative irradiation has been advocated, since it would still give a moderate degree of cell sterilization, but would cause little disturbance to the surgical field or to wound healing. Furthermore, it would be less damaging to adjacent normal tissue, and postoperative irradiation would still be possible if required (Nias, 1967).

It has been found that X-ray doses in excess of 1000 rad delay wound healing appreciably in C3H mice (Powers, 1965), and consequently it has been suggested that pre-operative single X-ray doses of 500–1000 rad should be selected.

At such low doses it was not thought necessary to give fractionated irradiation to spare the normal tissues, as is done in high-dose radiotherapy (Nias, 1967). However, two doses of X-ray might be more efficient than a single dose in sterilizing cells in the primary tumour, because reoxygenation and/or induced cell synchrony might cause the tumour to become more radiosensitive after the start of irradiation. Against this hypothetical advantage of 2 doses, should be set the delay between the first X-ray dose and the surgery, a delay in which further metastases might disseminate from a tumour incompletely sterilized by a low initial dose.

In the present work, two pre-operative radiation treatments were investigated: a single X-ray dose of 500 rad and two fractions of 350 rad given 24 h apart (chosen to produce approximately the same expected level of cell killing *in vitro* as the single dose). Surgical operation was carried out 24 h after the first X-ray dose in all cases.

### MATERIALS AND METHODS

The tumours studied were first-generation transplants of mammary carcinomas arising spontaneously in female mice, implanted s.c. on the anterior chest wall of male C3H/He mice from the Gray Laboratory inbred colony. The tumours have a volume doubling time of about 6 days from 6·5 to 8·2 mm mean diameter. The mice frequently develop secondary tumours in the lungs, but rarely elsewhere.

When the implanted tumours reached 6·5 $\pm$ 1 mm mean diameter (14–56 days after implant) the mice were randomized into one of three groups:

(1) a single dose of 500 rad plus a sham irradiation 24 $\pm$ 1 h later,
(2) two fractions of 350 rad given 24 $\pm$ 1 h apart,
(3) two sham irradiations given 24 $\pm$ 1 h apart.

Each group contained over 200 mice.

The X-irradiations were performed at 240kV and 15mA, using $\frac{1}{4}$ mm Cu + 1 mm Al filtration, to give a HVL of 1·3 mm Cu and a dose rate of 240 rad/min. The mice were placed in lead-shielded jigs, so that the tumours hung freely through a 2 $\times$ 2·5 cm oval hole. The mice breathed $O_2$ at 25 $\pm$ 1 °C during irradiation, so that the results could be compared with previous work (Sheldon *et al.*, 1974*a*). The scattered dose to the centre of the lungs was 22 rad per krad to the tumour.

I.p. injections of 60 mg/kg pentobarbitone sodium were used to anaesthetize the mice during irradiation. Whilst still under the anaesthetic for the second, real or sham irradiation, the tumours were excised surgically. This involved sterilizing the skin with 70% alcohol and, with the mouse supine, lifting up the tumour with forceps and cutting beneath it with curved scissors. Only one or two cuts were generally required to free the tumour, which in most cases adhered to the skin only and not to deeper tissue. The wound was closed using " autoclips " which were removed a fortnight later.

Bemigride, injected i.p. at a dosage of 0·5 mg/mouse, was given to revive the mice after either irradiation or surgery.

Manipulation of the tumours was constant for all groups. Three people performed the surgery and, although the number of mice done by each was different, the proportion in each of the 3 experimental groups was the same for each person.

The mice were sacrificed 10 weeks after surgery, as previous work had shown that by this time most of the mice that would develop lung metastases or local recurrences had already done so (Sheldon *et al.*, 1974*a*). The lungs were placed in Bouin's solution prior to assessment for pulmonary metastases.

Mice developing local recurrences (about 35%) were excluded from the analysis concerning metastases, as it would not be possible to determine whether metastases had been seeded from the original implanted tumour or from the subsequently recurring tumour. Mice with locally controlled tumours, which died prematurely from pulmonary metastases, were included in the analysis.

### RESULTS

The results were analysed in two ways: the effect of preoperative irradiations on success of surgery in locally controlling the tumours (Table I), and their influence on the development of distant metastases (Table II). The chi-squared test was used to evaluate differences.

TABLE I.—*The Failure Rate of Surgery in Local Control of Tumours* i.e. *the Number of Tumours which Recurred as a Percentage of the Number of Tumours Excised Surgically.* (*Actual Numbers in Parentheses.*)

| X-ray dose | % Total |
|---|---|
| 0 | 32 (68/212) |
| 500 | 37 (81/221) |
| 2 × 350 | 37 (80/216) |
| Total | 35 (229/649) |

TABLE II.—*The Incidence of Pulmonary Metastasis* i.e. *the Number of Mice with Macroscopic Metastases as a Percentage of the Number with Locally Controlled Tumours.* (*Actual Numbers in Parentheses.*)

| X-ray dose | % Total |
|---|---|
| 0 | 20 (29/144) |
| 500 | 25 (35/140) |
| 2 × 350 | 30 (41/135) |
| Total | 25 (105/420) |

The mean local failure rate (i.e. recurrence rate) was 32% after surgery and 37% after either of the preoperative irradiations. This was not a significant increase ($P = 0{\cdot}32$ and $0{\cdot}28$ using the chi-squared test). There was no significant difference between the results for the three operators.

The incidence of pulmonary metastasis was 20% (29/144) for the control mice receiving only sham irradiation, 25% (35/140) for those receiving a single dose of 500 rad, and 30% (41/135) for those receiving two fractions of 350 rad. The result for the 500 rad group was not significantly different from either the control group or the 2 fractions of 350 rad group. The latter group, however, had significantly more metastases than the control group ($P = 0{\cdot}048$). This was also true if the results for the operator doing the largest number of animals (150 + 138) were compared alone ($P = 0{\cdot}027$), but the difference was not significant for the other two operators (62 + 78 mice).

There was no significant difference between operators regarding the overall incidence of metastases.

### DISCUSSION

Several workers have tried to simulate the effect that preoperative irradiation has on the ability of cells disseminated at surgery to produce tumours, by studying the potential of previously irradiated tumour cells to produce tumours when implanted into new hosts (Hoye and Smith, 1961; Feder and Blair, 1964; Feder, Blair and Close, 1965). Not surprisingly they found that the larger the dose of radiation, the lower the probability of tumours subsequently developing. This is simply a reflection of the cell survival characteristics of mammalian cells, and not of the ability of a tumour *in situ* to disseminate its cells.

Our choice of a dose as low as 500 rad requires comment, in view of the higher doses required to cure 50% of the present tumours (~ 4700 rad) than of the lymphosarcomas of Powers and colleagues, or human squamous cell carcinoma nodules of comparable size (~ 2000 rad). The murine lymphosarcomas they used were strongly immunogenic, so that smaller doses of radiation should eradicate these tumours than in the case of non-immunogenic tumours. This could explain the difference between the two types of murine tumour. The X-ray doses required to cure murine tumours are almost certainly high because of the presence of hypoxic cells (Powers and Tolmach, 1964). Recent work on the present C3H tumours has shown that the " 50% cure dose " falls from 4700 to 2600 rad when the hypoxic-cell radiosensitizer Ro-07-0582 is administered shortly before irradiation (Sheldon, Foster and Fowler, 1974*b*). Thus the partial or complete elimination of hypoxic cells brings the X-ray cure dose into comparability with that for small human tumours. When low doses of 500 rad are used, the response of the tumour will be dominated by well-oxygenated cells (Powers and Tolmach, 1964) whose radiosensitivity would not be expected to be very different in men or mice. Thus our choice of 500 rad, while lower than the doses used by most but not all other experimenters, is not expected to be irrelevant to radiotherapy.

### *Local control*

In the present work, the success of surgery alone in locally controlling the tumour was 68% at 70 days, *i.e.* a 32% failure rate (Table I). This compares favourably with the success rates of other workers, *viz.* 57% at 100 days using s.c. implanted first-generation transplants of spontaneous mammary adenocarcinoma on the backs of mice (Inch and McCredie, 1963) and 53% for s.c. implanted 6C3HED tumours on the flanks of mice (Powers and Tolmach, 1964).

In the present work, the local success rates after surgery alone (68%) or preoperative irradiation with either one or two doses (63%) were not significantly different (Table I). The lack of difference

TABLE III.—*The Effect of Low-dose Pre-operative Irradiation on the Local Eradication of Tumours in Mice, Summarized According to Tumour Type. The Interval between Irradiation and Surgery was either Immediate or 1 day; Both intervals are Thought to Produce an equal Response (see text).*

| Tumour type | Tumour | Pre-operative dose (rad) | % Successful local control after Surgery alone | + Pre-op. irrad. | Interval between irrad. and surg. (days) | Authors |
|---|---|---|---|---|---|---|
| Lymphoma | 6C3HED | 500 | 53 | 85 | 0 | Powers & Tolmach, 1964 |
| | 6C3HED | 500 | 20 | 45 | 0 | Perez & Powers, 1967 |
| | 6C3HED | 500 | 20 | 45 | 1 | Perez & Powers, 1967 |
| | 6C3HED | 1000 | 20 | 65 | 0 | Perez & Powers, 1967 |
| | 6C3HED | 1000 | 20 | 65 | 1 | Perez & Powers, 1967 |
| | KHAA | 1000 | 30 | 66 | 0 | Powers & Palmer, 1968 |
| Melanoma | B16 | 1000 | 62 | 82 | 0 | Powers & Palmer, 1968 |
| | KHDD | 1000 | 46 | 67 | 0 | Powers & Palmer, 1968 |
| | | | $\bar{x} \sim 44$* | $\bar{x} \sim 69$* | | |
| Sarcoma | KHT | 1000 | 65 | 62 | 0 | Powers & Palmer, 1968 |
| | KHT | 1000 | 62 | 62 | 0 | Powers & Palmer, 1968 |
| Mammary Ca. | BW10232 | 1000 | 82 | 80 | 0 | Powers & Palmer, 1968 |
| | KHSH | 1000 | 100 | 100 | 0 | Powers & Palmer, 1968 |
| | C3H | 500 | 68 | 63 | 1 | Present work, 1976 |
| | C3H | $2 \times 350$ | 68 | 63 | 1α | Present work, 1976 |
| | | | $\bar{x} \sim 79$* | $\bar{x} \sim 79$* | | |

* mean for each tumour group.
α 24 h after first fraction, *i.e.* immediately after second fraction.

can be explained if each failure is the result of more cells being left behind at surgery than can be killed by 500 rad; this requires only about 10 cells if they are oxic. The local control rate had previously been found to be the same in a murine lymphosarcoma whether surgery followed radiation immediately or after delays of up to one week (Perez and Powers, 1967). Our results may therefore be compared with those of other animal studies using low-dose preoperative irradiation up to 1000 rad and short intervals up to one day. It should be remembered that the assumption that the interval is not critical remains to be justified for tumours other than the murine lymphosarcoma.

The results of the comparison with all the relevant published data are shown in Table III. Our results are not atypical, although our doses are lower than in most other work. Several murine mammary carcinomas and a sarcoma showed no improvement in local control, indeed a non-significant worsening occurs in some cases.

Various lymphosarcomas and melanomas, however, all showed a major improvement in local control. *This was because surgery alone gave poor results for the lymphosarcomas.* The argument is that an " optimum success rate " of 70% to 80% is provided by preoperative irradiation plus surgery, so that where surgery alone achieves nearly this rate, there is little more to gain by adding the radiation. Where surgery does not do well alone, preoperative radiation helps significantly, even at the low doses quoted. In support of this view, the average local control rates *with* preoperative low-dose irradiation were remarkably similar for the lymphomas and for the carcinomas and sarcoma. Thus low-dose irradiation helped for the tumours listed at the top of Table III but not for those at the bottom, which were well treated by surgery only in these mice.

Similarly, local control rates after *high-dose preoperative irradiation* were not significantly different for the two groups of tumours (lymphosarcomas versus other), or from the low dose results (compare Tables III and IV). This suggests that high-dose preoperative irradiation provides optimum treatments which, however, are not demonstrably better than those using low doses. Again, the results of surgery were poorer for the lymphoma group than for the carcinoma and sar-

TABLE IV.—*Review of the Effect of High-dose Pre-operative Irradiation on the Local Eradication of Tumours in Mice. The Interval between Irradiation and Surgery Varies from Immediate to Two Days.*

| Tumour type | Tumour | Pre-operative dose (rad) | % Successful local control after Surgery alone | + Pre-op. irrad. | Interval between irrad. and surg. (days) | Authors |
|---|---|---|---|---|---|---|
| Lymphoma | 6C3HED | 2000 | 20 | 80 | 0 | Perez & Powers, 1967 |
| | 6C3HED | 2000 | 20 | 77 | 1 | Perez & Powers, 1967 |
| | 6C3HED | 3000 | 20 | 84 | 1 | Perez & Powers, 1967 |
| | 6C3HED | 4000 | 20 | 80 | 1 | Perez & Powers, 1967 |
| | KHAA | 2000 | 33 | 80 | 0 | Powers & Palmer, 1968 |
| | KHAA | 2000 | 27 | 75 | 0 | Powers & Palmer, 1968 |
| | KHAA | 3000 | 10 | 44 | 0 | Powers & Palmer, 1968 |
| Hepatoma | MH134 | 2000 | 16 | 28 | 2 | Nakayama et al., 1963 |
| | | | $\bar{x} \sim 20$* | $\bar{x} \sim 58$* | | |
| Sarcoma | KHT | 3000 | 68 | 70 | 0 | Powers & Palmer, 1968 |
| Mammary Ca. | C3H/HeJ | 2000 | 57 | 66 | 1 | Inch & McCredie, 1963 |
| | | | $\bar{x} \sim 63$* | $\bar{x} \sim 68$* | | |

* Mean for each tumour group.

TABLE V.—*Effect of Pre-operative Irradiation on the Local Eradication of Walker Carcinoma in Rats. The Interval between the Last Irradiation and Surgery was not more than 24 h.*

| Tumour type | Pre-operative dose (rad) | % Successful local control after Surgery alone | Pre-op. irrad. | Authors | Comments |
|---|---|---|---|---|---|
| Walker 256 Ca-Sa (63 days) | Low dose 4 × 200 daily | 42%* (42/100) | 52% (52/100) | Agostino & Nickson, 1960 | Poor result with surgery only. |
| Walker 256 Ca-Sa (100 days) | High dose 2000 | 51% (18/35) | 88% (30/34) | Inch & McCredie, 1963 | Significant difference |

* Only 79 of the 100 unirradiated rats were suitable for operation, whereas in the irradiated group 90 of the 100 were suitable. Thus the radiation apparently reduced infiltration of the tumours, and helped them to be operable.

coma group, so that the advantage of preoperative irradiation was clearer for the former.

The two results that have been published for rat tumours are both for the antigenic Walker carcino-sarcoma (Agostino and Nickson, 1960; Inch and McCredie, 1963). They both responded poorly to surgery alone, and showed a significant improvement when preoperative radiation was used (Table V).

Thus no significant gain using preoperative radiotherapy was found for carcinomas or a sarcoma in mice, either in the present results or in those of other workers, because surgery alone appeared to be reasonably good. Preoperative radiation has been found, in the other work referred to, to be an advantage where the results of surgery alone were poor. High preoperative doses appeared to give a slightly more consistent advantage than low doses.

*Metastases*

In the present work, in mice whose tumours had been successfully locally controlled, the incidence of pulmonary metastases for those receiving no irradiation to the implanted tumour was 20%. For those receiving a single dose of 500 rad it was 25% and for those receiving two fractions of 350 rad it was 30%. The difference between the latter group and controls was just significant at the $P = 0{\cdot}05$ level. In the present work 30 animals per group were not sufficient to detect the difference, whereas 89–106

animals per group were sufficient (Table II).

Since the time interval between the first real or sham irradiation and surgical removal was constant at one day, such an increase could be caused if the first 350 rad irradiation led to a greater loss of viable metastatic cells from the tumour than with no irradiation; or if the second 350 rad dose led to greater spillage in the operation which immediately followed it. The cell killing effect of the two fractions of 350 rad was likely to be equal to, or even slightly greater than, that of the single dose of 500 rad, assuming that the tumour cells have radiosensitivities comparable with basal cells of the skin (Dutreix, Wambersie and Bounik, 1973; Douglas *et al.*, 1975). The effect of the first dose of 350 rad alone, however, would of course be less in sterilizing potentially metastasizing cells. The suggestion is that a first preoperative radiation dose should not be too low. It has been suggested that the anterior surface of the lung would have received more than 22 rad of scattered radiation measured at the centre of the body, and this may lead to the trapping of extra metastases den (Van Brenk *et al.*, 1973). However, this explanation is unlikely because, in another experiment, Sheldon (1974) showed, with tumours also transplanted on the anterior chest wall, that the number of metastases in each lobe of the lungs were simply proportional to the size of each lobe, for both irradiated and control mice. The chest wall site was used here for comparability with our earier work (Sheldon *et al.*, 1974*a*).

### CONCLUSIONS

Preoperative irradiation of transplanted mammary carcinomas in mice with low doses of X-rays produced no benefit in terms of reducing either local recurrences or distant metastases. On the contrary, the incidence of lung metastases in the group receiving two low doses of preoperative irradiation was just significantly higher (30%) than in the sham-irradiated controls (20%).

The present results agree with others in the literature for carcinomas and a sarcoma in mice. The benefit obtained by preoperative irradiation in previous work in murine lymphosarcomas seems to be because the poor local success with surgery only in that group of tumours leaves more room for improvement. Preoperative irradiation with either low or high doses has previously been shown to be advantageous for tumours in which the results of surgery only were poor. It helped to bring local control up to an optimum level. Clearly, further evidence is needed before generalizations can be attempted from one type of tumour to another.

We should like to thank the Cancer Research Campaign for support, Miss Sally Hill for expert technical assistance, Misses Angela Walder, Carol Dear and Mrs Suzanne Bull for breeding the mice, and Misses Ann Marriott and Jennifer Radmore for their subsequent care of the mice.

### REFERENCES

AGOSTINO, D. & NICKSON, J. J. (1960) Pre-operative X-ray Therapy in a Simulated Colon Carcinoma in the Rat. *Radiology*, **74,** 816.

DOUGLAS, B. G., FOWLER, J. F., DENEKAMP, J., HARRIS, S. R., AYRES, S. R., FAIRMAN, S., HILL, S. A., SHELDON, P. W. & STEWART, F. A. (1975) The Effect of Multiple Small Fractions of X-rays on Skin Reactions in the Mouse. *Proc. 6th L. H. Gray Conf. on The Initial Region of Cell Survival Curves.* Ed. T. Alper. (Inst. of Physics) p. 351.

DUTREIX, J., WAMBERSIE, A. & BOUNIK, C. (1973) Cellular Recovery in Human Skin Reactions: Application to Dose Fraction Overall Time Relationship on Radiotherapy. *Eur. J. Cancer*, **9,** 159.

FEDER, B. H. & BLAIR, P. B. (1964) Preoperative Irradiation: Evaluation by a Simple Experimental Model. *Radiology*, **83,** 111.

FEDER, B. H., BLAIR, P. B. & CLOSE, P. (1965) Fractionation in Preoperative Irradiation. *Radiology*, **84,** 447.

HOYE, R. C. & SMITH, R. R. (1961) The Effectiveness of Small Amounts of Preoperative Irradiation in Preventing the Growth of Tumour Cells Disseminated at Surgery. *Cancer, N.Y.*, **14,** 284.

INCH, W. R. & MCCREDIE, J. A. (1963) Effects of a Small Dose of X-radiation on Local Recurrence of Tumours in Rats and Mice. *Cancer, N.Y.*, **16,** 595.

NIAS, A. H. W. (1967) Radiobiological Aspects of Preoperative Irradiation. *J. Radiol.*, **40**, 166.

PEREZ, C. A. & POWERS, W. E. (1967) Studies on Optimal Dose of Preoperative Irradiation and Time for Surgery in the Case of a Mouse Lymphosarcoma. *Radiology*, **89**, 116.

POWERS, W. E. (1965) Radiation Biologic Considerations and Practical Investigation in Preoperative Radiotherapy. *J. Canad. Ass. Radiol.*, **16**, 217.

POWERS, W. E. & PALMER, L. A. (1968) Biologic Basis of Preoperative Radiation Treatment. *Am. J. Roent.*, **102**, 176.

POWERS, W. E. & TOLMACH, L. J. (1964) Preoperative Radiation Therapy: Biological Basis and Experimental Investigation. *Nature, Lond.*, **201**, 272.

SHELDON, P. W. (1974) The Effect of Irradiating a Transplanted Solid Sarcoma on the Subsequent Development of Metastases. *Br. J. Cancer*, **30** 416.

SHELDON, P. W., BEGG, A. C., FOWLER, J. F. & LANSLEY, I. F. (1974*a*) The Incidence of Lung Metastases in C3H Mice after Treatment of Implanted Solid Tumours with X-rays or Surgery. *Br. J. Cancer*, **30**, 342.

SHELDON, P. W., FOSTER, J. L. & FOWLER, J. F. (1974*b*) Radiosensitization of C3H mouse mammary Tumours by a 2-Nitroimidazole Drug. *Br. J. Cancer*, **30**, 560.

VAN DEN BRENK, H. A. S., BURCH, W. M., ORTON, C. & SHARPINGTON, C. (1973) Stimulation of Clonogenic Growth of Tumour Cells and Metastases in the Lungs by Local X-radiation. *Br. J. Cancer*, **27**, 291.