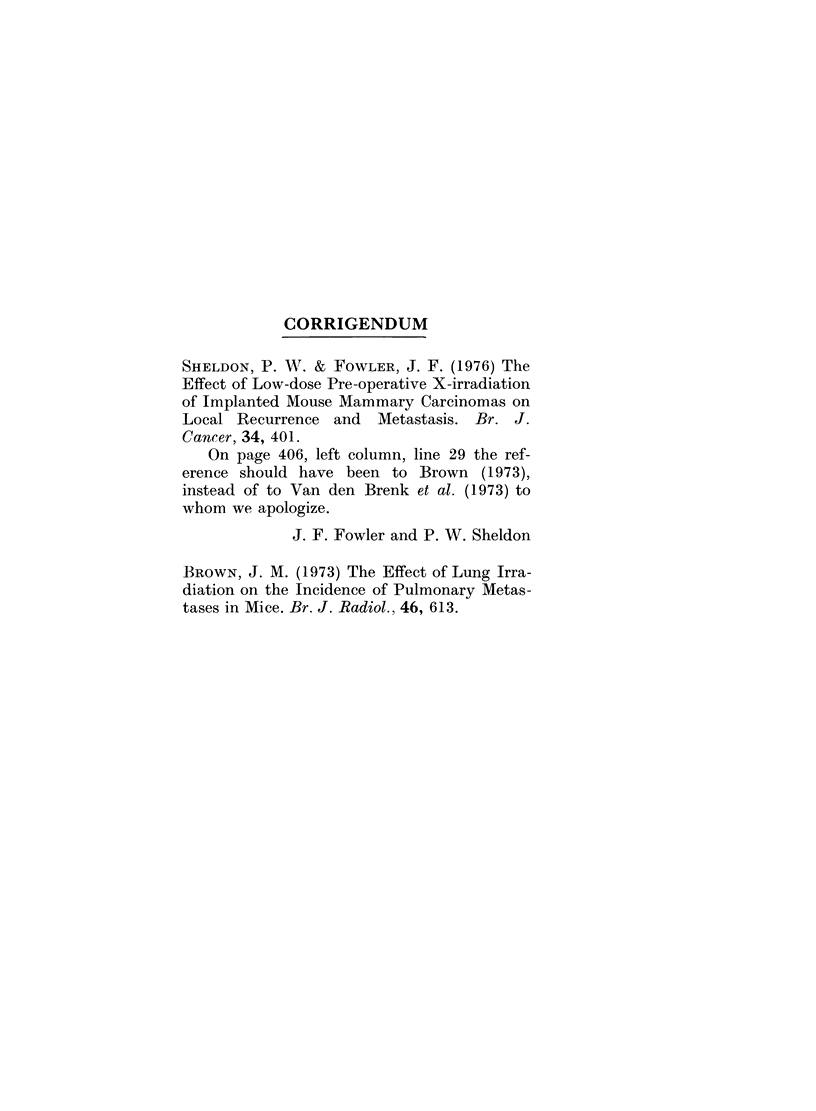# Corrigendum

**Published:** 1976-10

**Authors:** 


### CORRIGENDUM

Sheldon, P. W. & Fowler, J. F. (1976) The Effect of Low-dose Pre-operative X-irradiation of Implanted Mouse Mammary Carcinomas on Local Recurrence and Metastasis. *Br. J. Cancer*, **34,** 401.

On page 406, left column, line 29 the reference should have been to Brown (1973), instead of to Van den Brenk *et al.* (1973) to whom we apologize.

J. F. Fowler and P. W. Sheldon